# Detection of a CO and NH_3_ gas mixture using carboxylic acid-functionalized single-walled carbon nanotubes

**DOI:** 10.1186/1556-276X-8-12

**Published:** 2013-01-04

**Authors:** Ki-Young Dong, Jinnil Choi, Yang Doo Lee, Byung Hyun Kang, Youn-Yeol Yu, Hyang Hee Choi, Byeong-Kwon Ju

**Affiliations:** 1 Display and Nanosystem Laboratory, School of Engineering, Korea University, 5-1 Anam-Dong, Seongbuk-Gu, Seoul, 136-713, Republic of Korea; 2 Department of Materials Science and Engineering, Yonsei University, Seoul, 120–749, Republic of Korea; 3 Department of Mechanical Engineering, Hanbat National University, Daejeon, 305-719, Republic of Korea

**Keywords:** Gas sensors, Carboxylic acid functionalized, Single-walled carbon nanotubes, Mixture gas

## Abstract

Carbon nanotubes (CNT) are extremely sensitive to environmental gases. However, detection of mixture gas is still a challenge. Here, we report that 10 ppm of carbon monoxide (CO) and ammonia (NH_3_) can be electrically detected using a carboxylic acid-functionalized single-walled carbon nanotubes (C-SWCNT). CO and NH_3_ gases were mixed carefully with the same concentrations of 10 ppm. Our sensor showed faster response to the CO gas than the NH_3_ gas. The sensing properties and effect of carboxylic acid group were demonstrated, and C-SWCNT sensors with good repeatability and fast responses over a range of concentrations may be used as a simple and effective detection method of CO and NH_3_ mixture gas.

## Background

Among several applications using carbon nanotubes (CNT) [1], chemical gas sensors are currently regarded as one of the most promising application due to their fast response and high sensitivity toward gaseous molecules at low operational temperatures. Although considerable theoretical efforts have been devoted to the study of the possible interaction of a broad variety of gas molecules including H_2_, NH_3_, NO_2_, O_2_, and CO with CNT [2-9], these gases are frequently found in the polluted air from modern big cities. Therefore, to commercialize gas sensors using CNT as sensing materials, sensing experiments should be performed in a mixed gas environment in order to take actual air characteristics into account. Sensing mixture-gas molecules is important for environmental monitoring, control of chemical processes, agriculture, and biological and me2dical applications. Upon exposure to gas molecules, the electrical resistance of single-walled carbon nanotubes (SWCNT) changes and the threshold voltage is shifted due to charge transfer between the semiconducting SWCNT and electron-withdrawing and electron-donating molecules. Theoretical calculations showed the binding energy of CO and NH_3_ to SWCNT, which indicate a weak charge transfer. The conductivity change may also be caused by contact between the electrode and SWCNT, and the contact between SWCNT [8-11]. Both CO and NH_3_ are toxic, and even a small amount of exposure for a given period could lead to fatality, where detection of the former can be difficult due to its characteristics, having no odor and color, while the latter becomes dangerous for the environment in an anhydrous state, flammable, and can form explosive mixtures with air, especially for agricultural industries [12-14]. The detection of the CO and NH_3_ gases was reported by Fu et al. and Kong et al., respectively [7,15]. They suggested that the sensing characteristics of the CO and NH_3_ gases by carbon nanotubes are different for each gas. Additionally, CO gas molecules are adsorbed on carboxylic acid functionalities through weak hydrogen bonding, while the NH_3_ molecules are physically and chemically adsorbed and work as an electron donor like H_2_O [16]. In this study, to incorporate mixture of gases as well as individual detection, a gas sensor using carboxylic acid-functionalized single-walled carbon nanotubes (C-SWCNT) was introduced for CO and NH_3_ gases. Also, comparisons will be made with conventional sensors highlighting improved characteristics.

## Methods

High-purity SWCNT, purchased from Hanwha Nanotech, Inc. (Incheon, South Korea), are synthesized by the arc-discharge method, with purity of about 90%. The SWCNT have diameters between 1 and 1.2 nm and were very long (5 to 15 μm). For the experiments in this research, 100 mg of SWCNTs were dispersed in 100-mL deionized water (DI) water and sonicated for 2 h using bath sonicator (frequency 53 kHz, power 180 W). Then, nitric acid was added to the dispersion to reach 6 M acid concentration for highly carboxylic acid group functionalized. This dispersion was further sonicated for 4 h. The dispersion was filtered through polytetrafluoroethylene (PTFE) membrane (pore diameter 450 nm) and repeatedly washed with DI water. The resulting C-SWCNT film was easily peeled off from the PTFE membrane. The control C-SWCNT film was formed by filtering the aqueous C-SWCNT dispersion without nitric acid that has been sonicated for 6 h. The films were dried at 80°C in a vacuum and heat-treated in air at 200°C for 2 h. Then, the tube solution consisted of approximately 32 mg/L of individual C-SWCNT in a 0.6 wt% aqueous sodium dodecyl sulfate (SDS) solution. The C-SWCNTs were dispersed in DI water with the SDS which is used to obtain a stable colloidal suspension of C-SWCNTs. Dispersion of C-SWCNT was performed in a bath sonicator for 6 h and then centrifuged for 30 min at 4,500 rpm. This method is simple and classically employed to disperse C-SWCNT in deionized water with the help of commercially available SDS molecules [17]. The steric repulsion force introduced by the surfactant overcomes the van der Waals attractions between the SDS-wrapped C-SWCNT surfaces. Wrapping nanotubes with SDS surfactant guarantees that tubes previously separated by sonication will no rejoin [18].

**Figure 1:**
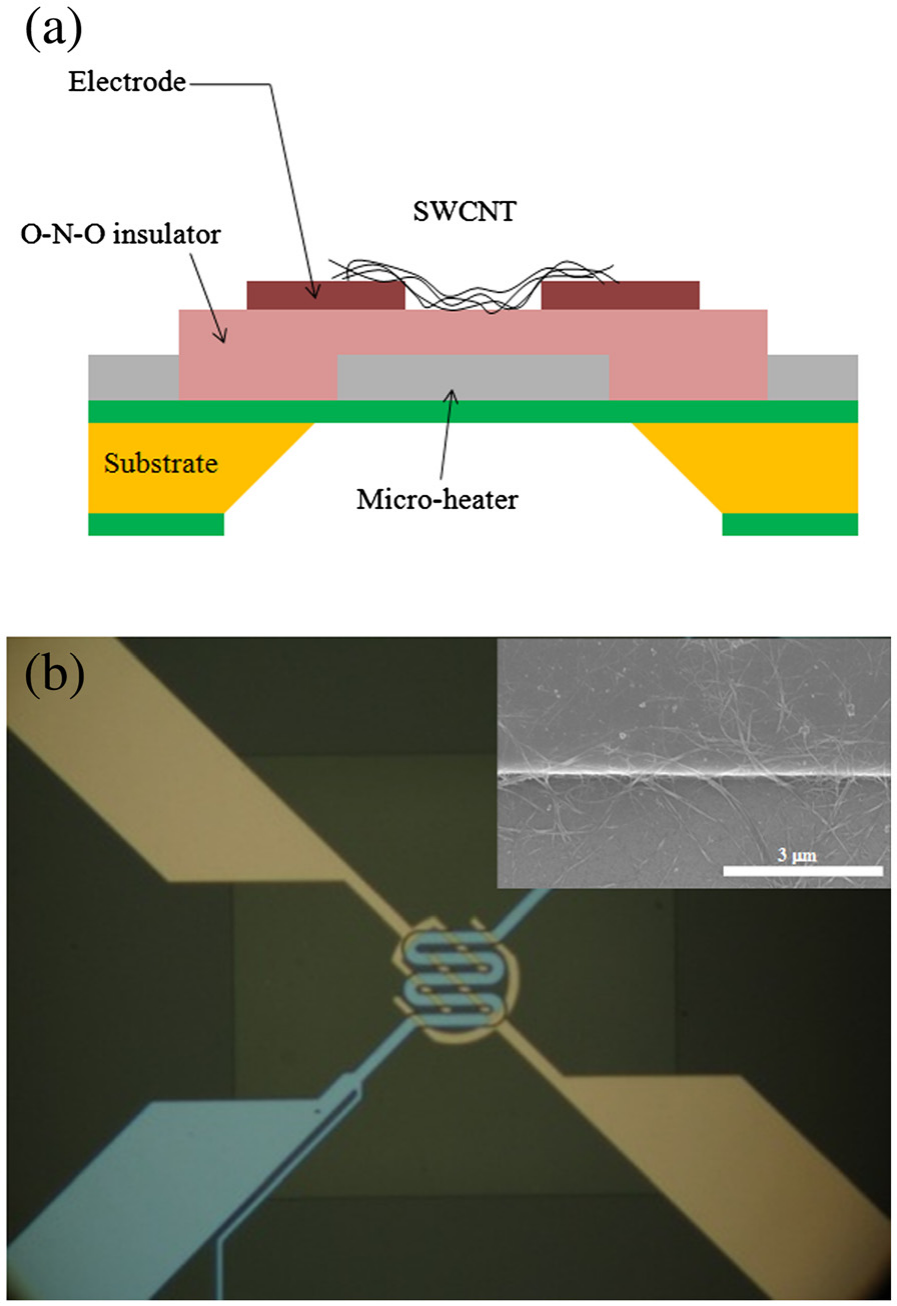
**Interdigitated electrodes and fabricated gas sensor.** (**a**) The interdigitated electrodes. (**b**) An optical image of the fabricated gas sensor. Inset is a SEM image of C-SWCNT after drying across the electrode on a bare surface. Detection of a CO and NH_3_ gas mixture using carboxylic acid-functionalized single-walled carbon nanotubes.

The schematic of our sensor is shown in Figure 1a. The device, integrated with a micro-heater, was fabricated on Si wafer with all of the patterning processes performed by photolithography. Initially, a low-stress SiN_*x*_ layer was deposited on the wafer using low pressure chemical vapor deposition. In order to create the micro-heater, Ti/Pt were then deposited by e-beam evaporation and patterned. An oxide-nitride-oxide layer was deposited by plasma-enhanced chemical vapor deposition to provide electrical insulation between the electrode and the micro-heater. As for the electrodes, Ti/Au were deposited by sputtering and then patterned. In addition, the backside of the silicon was etched by a KOH etchant to generate thermally insulated heater membranes. Finally, the well-dispersed C-SWCNT solution was dropped onto the wafer. The wafer was then heated in an oven at 220°C for 20 min to remove the SDS. An optical image of the fabricated MEMS gas sensor is shown in Figure 1b.

We experimentally found that the resistances of the C-SWCNT typically ranged from 4 to 5 kΩ, depending on the amount of C-SWCNT across the electrode pair. The flow rate of N_2_ and the concentration of gases (CO, NH_3_, and their mixture) were controlled by pneumatic mass flow controllers. The resistance change value was measured and stored by a source meter (Keithley 2400, Keithley Instruments, Inc., Cleveland, USA) and LabVIEW (National Instrument Corp., Austin, USA) software, respectively. Adsorbed gases were desorbed-vent with N_2_ flow.

## Results and discussion

In our experiment, the sensor response was evaluated by measuring the resistance upon exposure to various gases. The sensor response is defined as

(1) $$\mathrm{\Delta R}=\frac{R_{g}-R_{0}}{R_{0}}Χ100$$

where *R*_g_ represents the resistance upon exposure to the test gases, and *R*_0_ is the initial resistance in the presence of N_2_. The carrier gas (N_2_) flux was maintained at 500 sccm throughout the experiment.

**Figure 2:**
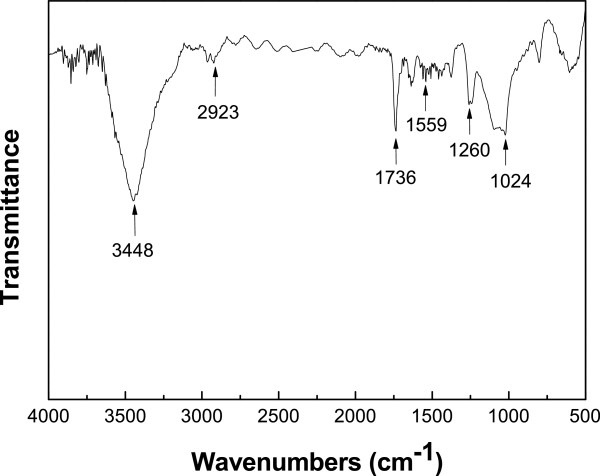
**FT-IR spectra of the C-SWCNT.** Detection of a CO and NH_3_ gas mixture using carboxylic acid-functionalized single-walled carbon nanotubes.

Figure 2 is the FT-IR spectrum of C-SWCNT, which shows the C=O stretching of the -COOH group and a very broad O-H stretching peak from 3,100 to 3,600 cm^−1^. The peaks at 1,024 and 2,923 cm^−1^ can be assigned to C-OH stretch mode and C-H stretch mode in methane, respectively. The peaks of COOH and COO^−^ at 1,736 and 1,559 cm^−1^ were also present.

**Figure 3:**
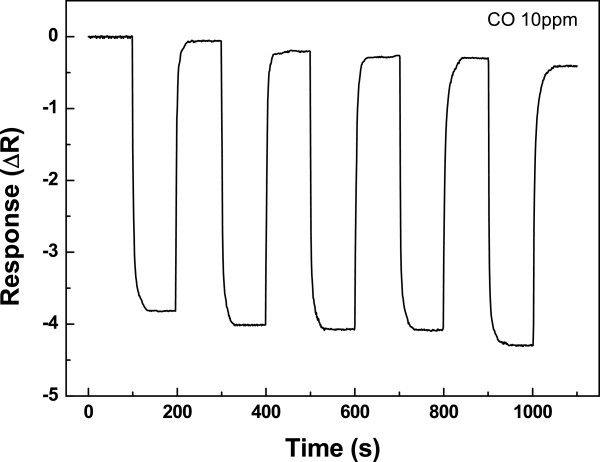
**Electrical resistance changes at 150°C with 10 ppm of CO.** Electrical resistance changes of the sensor as a function of time for five cycles at 150°C with 10 ppm of CO. Detection of a CO and NH_3_ gas mixture using carboxylic acid-functionalized single-walled carbon nanotubes.

Figure 3 shows the fast response and recovery times recorded during five short exposures to the 10 ppm CO gas at 150°C. Since the pristine SWCNT gas sensor was insensitive to CO gas due to the low affinity to pristine SWCNT [19], we considered that highly C-SWCNT was responsible for the observed decrease in resistance under CO gas. The change in resistance is suspected from the interaction between CO gas and the carboxylic acid group on C-SWCNT sidewalls. It has been reported that the CO gas can be absorbed on carboxylic acid functionalities through weak hydrogen bonding [6-8,16]. Consequently, the carboxylic acid group functionality may play a key role in CO gas detection, resulting in a decrease in the electrical resistance of C-SWCNT despite the interaction with the electron-withdrawing gas. Electron withdrawing due to the carboxylic acid group on the sidewalls will transfer electrons to C-SWCNT, thereby giving more hole carriers to the C-SWCNT. Therefore, it is reasonable to consider that the carboxylic acid groups introduced on the surface caused an enhancement of charge density in the C-SWCNT and, hence, increased the amount of electron transfer between C-SWCNT and CO molecules, which increased the hole current of p-type C-SWCNT.

**Figure 4:**
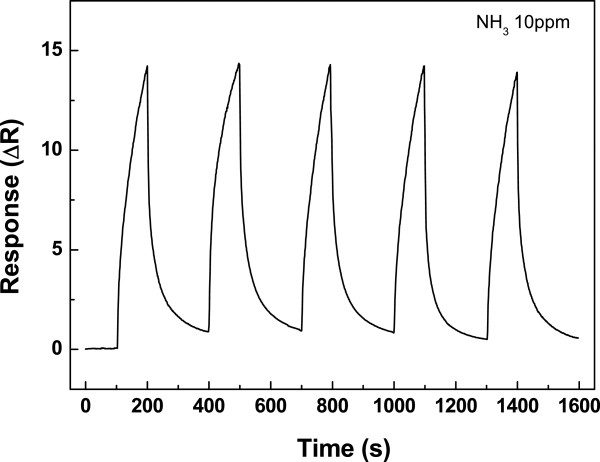
**Electrical resistance changes at 80°C with 10 ppm of NH**_**3**_**.** Electrical resistance changes of the sensor as a function of time for five cycles at 80°C with 10 ppm of NH_3_. Detection of a CO and NH_3_ gas mixture using carboxylic acid-functionalized single-walled carbon nanotubes.

Figure 4 demonstrates the time dependence of C-SWCNT resistance when exposed to 10 ppm NH_3_ gas at 80°C. The increase of the resistance can be explained as the following: since it is known that each NH_3_ molecule has a lone electron pair that can be donated to other species, therefore, NH_3_ is a donor gas. When the sensor is exposed to NH_3_ molecules, electrons are transferred from NH_3_ to C-SWCNT. NH_3_ donates electrons to the valence band of the C-SWCNT, which leads to the increase in electrical resistance of sensors due to the reduced number of hole carriers in the C-SWCNT. The increase in resistance is an evidence that the SWCNT is a p-type semiconductor.

We conducted an experiment to get the response of the mixed gas consisting of electron-withdrawing and electron-donating gases. One gas had a faster response time and lower sensor response than the other. In our experiment, CO and NH_3_ were chosen as gases having a faster response time with weak bonding and faster sensor response with strong bonding, respectively. Previous studies reported individual detection of CO [6-8,20] and NH_3_[14], where these sensors were using C-SWCNT bundle sensing layer, accordingly. As well as introducing mixture-gas detection capability, the C-SWCNT sensor fabricated in our study was more responsive even for individual detection, see Figures 3 and 4.

**Figure 5:**
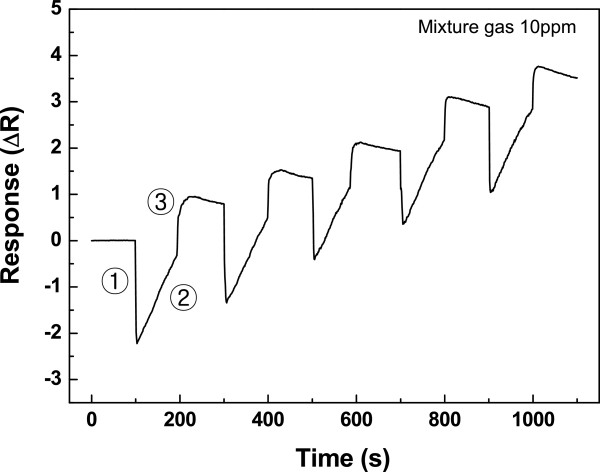
**The electrical resistance changes (150°C with 10 ppm of a CO and NH**_**3**_**gas mixture).** The electrical resistance changes of the sensor as a function of time for five cycles at 150°C with 10 ppm of a CO and NH_3_ gas mixture. Detection of a CO and NH_3_ gas mixture using carboxylic acid-functionalized single-walled carbon nanotubes.

Figure 5 indicates the sensing result of the gas mixture of CO and NH_3_ at 150°C. Exposure to the gas mixture rapidly decreased and increased the resistance of the C-SWCNT network. Similar behavior had been observed with individual C-SWCNT sensors. Repetitive cycles are observed, and therefore, one cycle will be explored. At point ①, the resistance was decreased due to the initial CO reaction with the surface of the C-SWCNT carboxylic acid group in the gas mixture. As the physical and chemical reactions between NH_3_ and CO progressed, the resistance was increased gradually in the gas mixture at point ②. Then, at point ③, a sharper increase in the resistance was observed as new gas was produced from the chemical reaction. The decrease of resistance in a cycle may be due to the adsorption of CO, because the response of the CO was faster than that of the NH_3_ at point ①. Finally, the resistance was increased more than the initial value. This seems to be because the C-SWCNT had a higher sensor response to NH_3_ than to the CO adsorbed into the C-SWCNT later at point ②.

**Figure 6:**
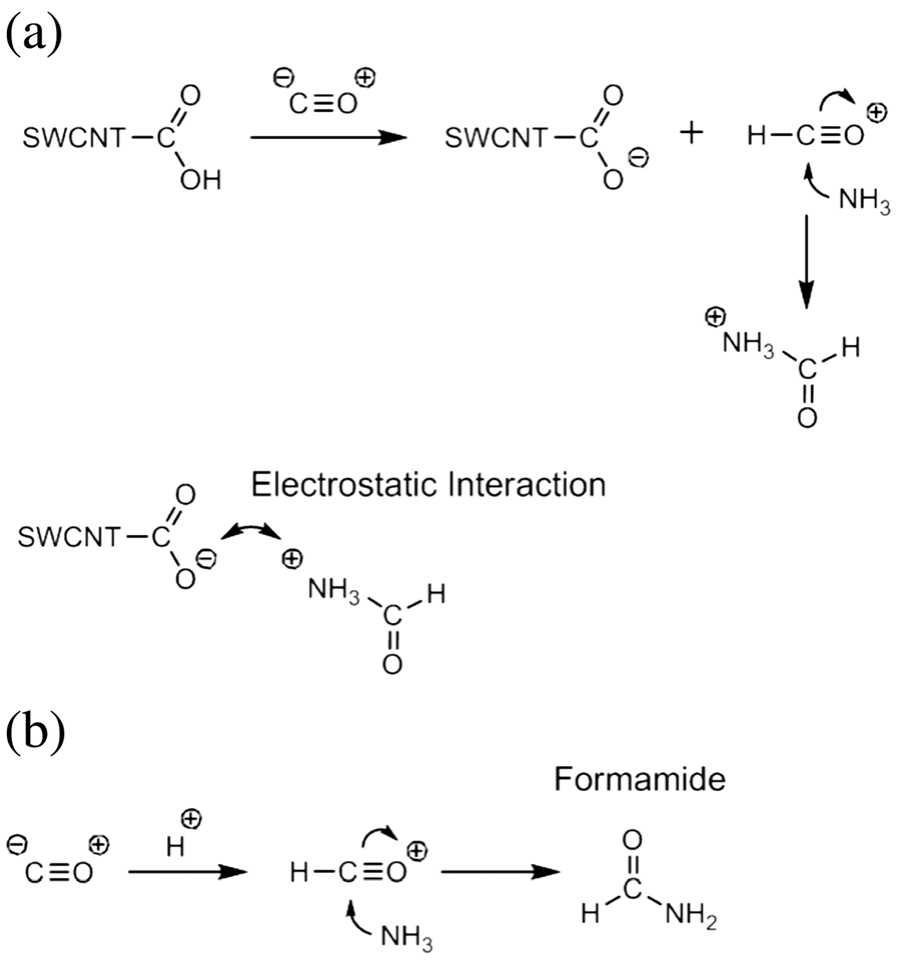
**The mechanism of the gas mixture's chemical reaction.** The mechanism when (**a**) the surface of the C-SWCNT consists of -COOH. (**b**) The surface of the C-SWCNT consists of -COO or -OH at 150°C. Detection of a CO and NH_3_ gas mixture using carboxylic acid-functionalized single-walled carbon nanotubes.

Figure 6a shows the expected reaction in the case of the gas mixture of CO and NH_3_. When the two gases, CO as the acceptor gas and NH_3_ as the donor gas, are mixed in the same volume, a nucleophilic addition occurs. The main acidic functionalities comprise carboxylic (−COOH), carbonyl (−C=O), and hydroxide (−OH) groups [21] approximately in a proportion of 4:2:1 [22] on the surface of C-SWCNT. CO and NH_3_ gases, being basic, react with sub-acidic -COOH but not with -C=O and neutral -OH, respectively. When the surface of the C-SWCNT consists of -COOH as shown in Figure 6a, the CO gas reacts with the hydrogen (H) of -COOH initially. Then NH_3_ is introduced to the reaction, resulting in a nucleophile attack on the carbon. From these reactions, positive charge is transferred to the surface of the gas mixture's molecules. Therefore, negative charge is formed on the surface of the C-SWCNT by losing H from -COOH. The resulting -COO- charge on the C-SWCNT surface is then bonded with the gas mixture by electrostatic interaction. These chemical reactions seemed to be a factor for the changes in the electronic characteristics as shown in Figure 5 at point ③. In contrast, when the surface of C-SWCNT consists of -C=O or -OH, C-SWCNT and gas molecules do not react and, therefore, form a formamide as shown in Figure 6b. The N_2_ gas, which did not participate in the reaction, was introduced continuously into the inside of the chamber where the reaction of the gases was highly anhydrous.

**Figure 7:**
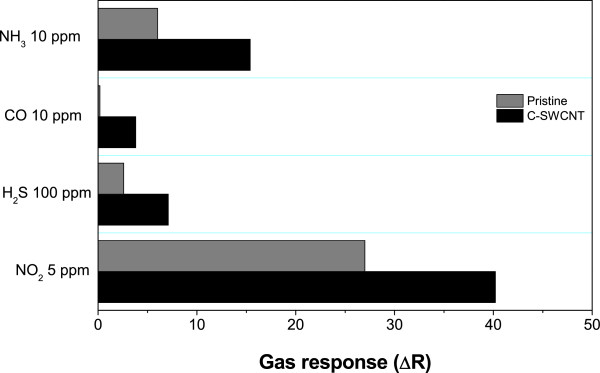
**Gas response of the pristine and C-SWCNT gas sensors showing the selectivity for different gases.** Detection of a CO and NH_3_ gas mixture using carboxylic acid-functionalized single-walled carbon nanotubes.

For practical use, the selectivity of the gas sensors is also an important consideration. A comparison between the responses of the sensors for different gases is shown in Figure 7. It is found that the C-SWCNT exhibits larger response at all gases. It is clear that the C-SWCNTs are highly selective to gases.

## Conclusion

The C-SWCNT-based sensor was used to detect the change of resistance when the sensor was exposed to three types of gases. When the sensor was exposed separately to CO and NH_3_, the sensor resistance decreased and increased, respectively. However, in the case of a mixture of the gases, CO and NH_3_, the resistance was decreased due to an initial reaction of CO with the surface of the C-SWCNT in the gas mixture. The decrease of resistance in a cycle may be due to the adsorption of CO because the response of the CO was faster than that of the NH_3_. As the chemical reaction between NH_3_ and CO progressed, the resistance was gradually increased.

However, since we presume that the absorption on CO is much faster than that on NH_3_, absorbed CO gas firstly reacts with the C-SWCNT, followed by the reaction of NH_3_ gas which has a dominant and proper reaction in the total reaction. A comparison was made with conventional sensors, showing enhanced sensor response for individual detection. Also, selectivity for mixture-gas detection was explored, and this result clearly shows that a C-SWCNT-based gas sensor can be a good candidate for mixture-gas detection.

## Competing interest

The authors declare that they have no competing interests.

## Authors' contributions

The work presented here was carried out in collaboration among all authors. KYD, HHC, and BKJ defined the research theme. KYD, JC, and YDL designed the methods and experiments, carried out the laboratory experiments, analyzed the data, interpreted the results, and wrote the paper. BHK and YYY worked on the associated data collection and their interpretation, and wrote the paper. KYD, HHC and BKJ designed the experiments, discussed the analyses, and wrote the paper. All authors read and approved the final manuscript.
